# SWI/SNF regulates half of its targets without the need of ATP-driven nucleosome remodeling by Brahma

**DOI:** 10.1186/s12864-018-4746-2

**Published:** 2018-05-18

**Authors:** Antonio Jordán-Pla, Simei Yu, Johan Waldholm, Thomas Källman, Ann-Kristin Östlund Farrants, Neus Visa

**Affiliations:** 1 Department of Molecular Biosciences, The Wenner-Gren Institute, Stockholm University, SE-106 91 Stockholm, Sweden; 2 Department of Medical Biochemistry and Microbiology, Uppsala University, SE-751 23 Uppsala, Sweden

**Keywords:** SWI/SNF, Gene expression, Transcription regulation, *Drosophila melanogaster*

## Abstract

**Background:**

Brahma (BRM) is the only catalytic subunit of the SWI/SNF chromatin-remodeling complex of *Drosophila melanogaster*. The function of SWI/SNF in transcription has long been attributed to its ability to remodel nucleosomes, which requires the ATPase activity of BRM. However, recent studies have provided evidence for a non-catalytic function of BRM in the transcriptional regulation of a few specific genes.

**Results:**

Here we have used RNA-seq and ChIP-seq to identify the BRM target genes in S2 cells, and we have used a catalytically inactive BRM mutant (K804R) that is unable to hydrolyze ATP to investigate the magnitude of the non-catalytic function of BRM in transcription regulation. We show that 49% of the BRM target genes in S2 cells are regulated through mechanisms that do not require BRM to have an ATPase activity. We also show that the catalytic and non-catalytic mechanisms of SWI/SNF regulation operate on two subsets of genes that differ in promoter architecture and are linked to different biological processes.

**Conclusions:**

This study shows that the non-catalytic role of SWI/SNF in transcription regulation is far more prevalent than previously anticipated and that the genes that are regulated by SWI/SNF through ATPase-dependent and ATPase-independent mechanisms have specialized roles in different cellular and developmental processes.

**Electronic supplementary material:**

The online version of this article (10.1186/s12864-018-4746-2) contains supplementary material, which is available to authorized users.

## Background

SWI/SNF is an evolutionarily conserved ATP-dependent chromatin remodeling complex that controls fundamental biological processes such as progress through the cell cycle, development and metabolism [1, 2]. SWI/SNF uses ATP hydrolysis to remodel nucleosomes at promoter regions and within gene bodies, and SWI/SNF is an important player in transcription regulation [3–5]. Brahma (BRM) is the only ATPase subunit of the SWI/SNF complex of *Drosophila melanogaster.* The catalytic activity of the mammalian SWI/SNF is provided by one of two paralogs BRM and BRM-related gene 1 (BRG1).

Much work has been directed towards identifying BRM target genes, and ChIP-seq studies have provided genome-wide maps of SWI/SNF occupancy in different organisms including nematodes [6], mammals [7, 8] and plants [9, 10]. SWI/SNF binds to promoters, enhancers and insulator regions in the mammalian genome [7], and it is recruited to its genomic targets through interactions with post-translationally modified histones and chromatin proteins [11]. However, much remains to be understood about the mechanisms by which SWI/SNF regulates gene expression.

In recent years, experiments in human and fly cells have provided evidence that SWI/SNF regulates not only transcription but also pre-mRNA processing, and that this role at the RNA level is uncoupled from the nucleosome-remodeling capacity of SWI/SNF [12–16]. The regulation of gene transcription by SWI/SNF has instead been regarded as a process that is intimately associated with the ability of BRM to undergo conformational changes in an ATP-dependent manner. In mammals, an intact ATPase domain of either BRM or BRG1 is essential for the nucleosome-remodeling activity of the SWI/SNF complex in vitro and in vivo [17–21]. However, at least two independent studies have suggested that transcription regulatory events may take place in *D. melanogaster* that involve BRM but not its ATPase activity. In one of the studies*,* Zraly and coworkers proposed that BRM represses the expression of the late *Eig* genes of *D. melanogaster* through a mechanism that does not require a functional ATPase domain [22]. In a more recent report, Kwok and coworkers showed that BRM fine-tunes circadian transcription in a non-catalytic manner by recruiting transcriptional repressors to target genes [23]. These observations prompted us to investigate the extent of the ATPase-independent function of BRM in transcription regulation. We combined RNA-seq and ChIP-seq to identify BRM target genes in S2 cells, and we made use of the dominant-negative mutant *Brm*^*K804R*^ that lacks ATPase activity [20] to study the magnitude of the non-catalytic roles of SWI/SNF in transcription regulation on a genomic scale. We show that approximately half of the BRM target genes in S2 cells (49%) are regulated through mechanisms that do not require BRM to have an ATPase activity, which reveals that the non-catalytic mode of action is far more prevalent than previously anticipated. The groups of genes regulated by BRM through catalytic and non-catalytic mechanisms are involved in different biological processes and are characterized by different promoter configurations, which reveals an additional level of complexity in the mechanisms of SWI/SNF action.

## Results

### The BRM-bound genes in the genome of S2 cells

**Fig. 1:**
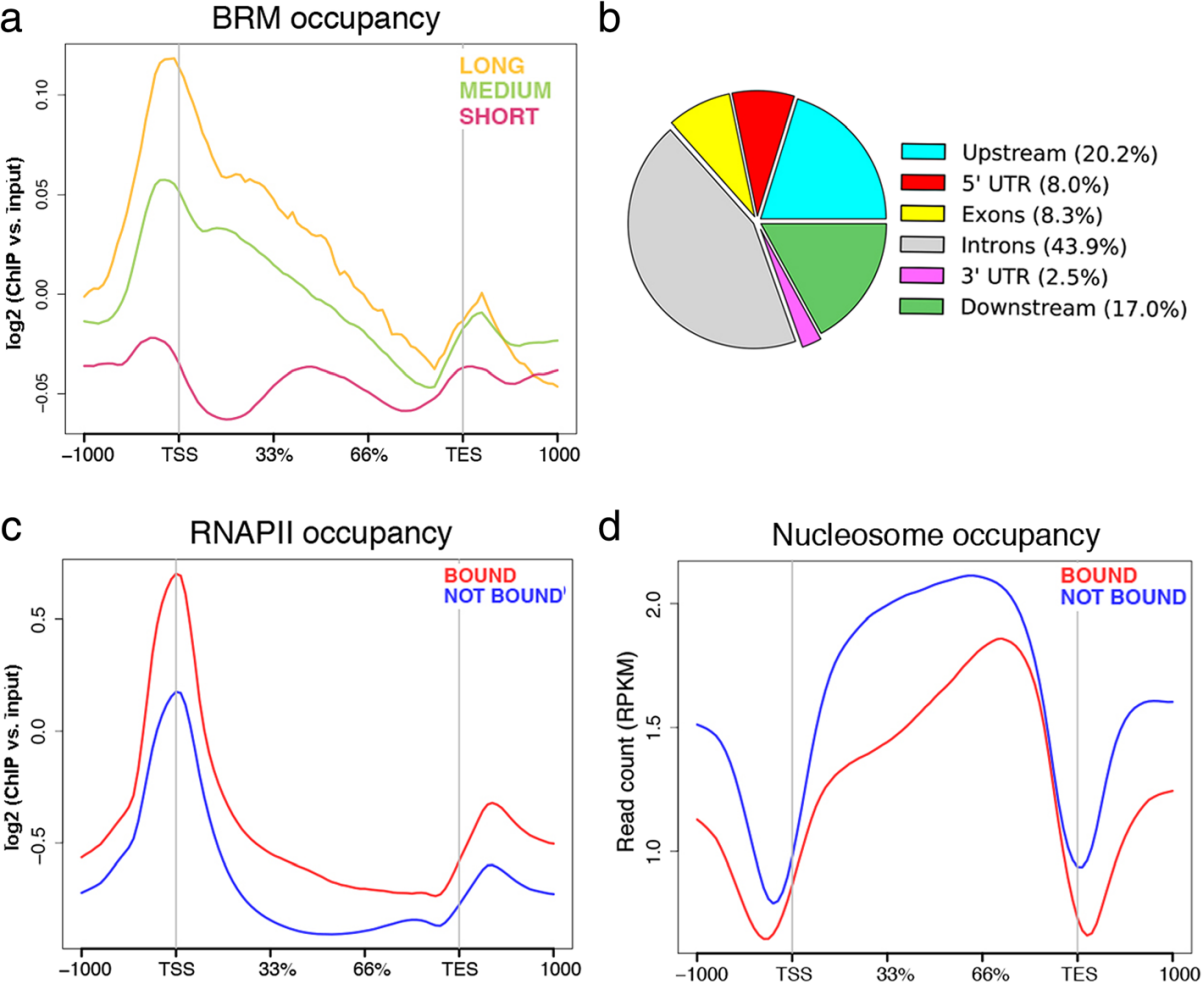
BRM occupancy in S2 cells analyzed by ChIP-seq with an antibody against the endogenous BRM. **a** The average distribution of BRM in genes classified according to gene length as indicated in the figure. *n* = 1357, 7763 and 4798 for long, medium and short genes, respectively. **b** The pie diagram shows the distribution of BRM peaks in the genome of S2 cells in relation to gene features. **c** Metagene analysis of RNAPII distribution in the BRM-bound (red, *n* = 2521) and not bound (blue, *n* = 11,403) genes. The plot is derived from data from Lam et al. [52]. The antibody used for the RNAPII ChIP-seq experiment was directed against Rpb3. **d** The average nucleosome density in the BRM-bound and not bound genes. The plot is derived from data from Shi et al. [25]

ChIP-seq experiments using an antibody against the endogenous BRM identified 2521 genes bound by BRM in S2 cells of *D. melanogaster*. BRM binds upstream of the transcription start site (TSS) and downstream of the cleavage and polyadenylation site (also referred to as “transcription end site” or TES). Moreover, a substantial amount of BRM is bound to the gene bodies (Fig. 1a-b), in agreement with reports from other species [4, 8]. The abundance of BRM in introns was surprisingly high (43.9%, Fig. 1b). Introns were also highly represented (28%) among the BRG1-enriched regions identified by Attanasio and coworkers in human cells [8].

On average, the BRM-bound genes in S2 cells were characterized by higher levels of RNA polymerase II (RNAPII) than the rest of the genes (Fig. 1c). This observation is in good agreement with previous studies showing that BRM associates preferentially with transcriptionally active loci in the polytene chromosomes of *D. melanogaster* [24] and *Chironomus tentans* [13]. However, the levels of BRM were not correlated with the levels of RNAPII at individual genes (Additional file 1: Figure S1), in agreement with the fact that many BRM target genes are repressed by BRM (see for example reference [3] and our results below). Moreover, the BRM-bound genes were characterized by a much lower nucleosome density in the gene body than the unbound genes, and by more pronounced nucleosome-depleted regions upstream and downstream of the TSS and TES, which can be seen by comparing our ChIP-seq results with results from a micrococcal nuclease study (MNase-seq) carried out by Shi et al. [25] (Fig. 1d). Interestingly, BRM was preferentially associated with long genes (> 15 kb) and was underrepresented in short genes (< 1.5 kb) (Fig. 1a). The presence of BRM in gene bodies and its preferential association with long genes suggests that SWI/SNF plays a prominent role in transcription elongation. The presence of BRM in gene bodies may also be linked to its role in pre-mRNA splicing [12, 13]. Roles in RNAPII elongation have been attributed to Swi2 in budding yeast [4] and BRG1 in mammals [5], and may provide a mechanistic basis for the regulation of alternative splicing by SWI/SNF [12].

### Identification of BRM target genes in S2 cells

**Fig. 2:**
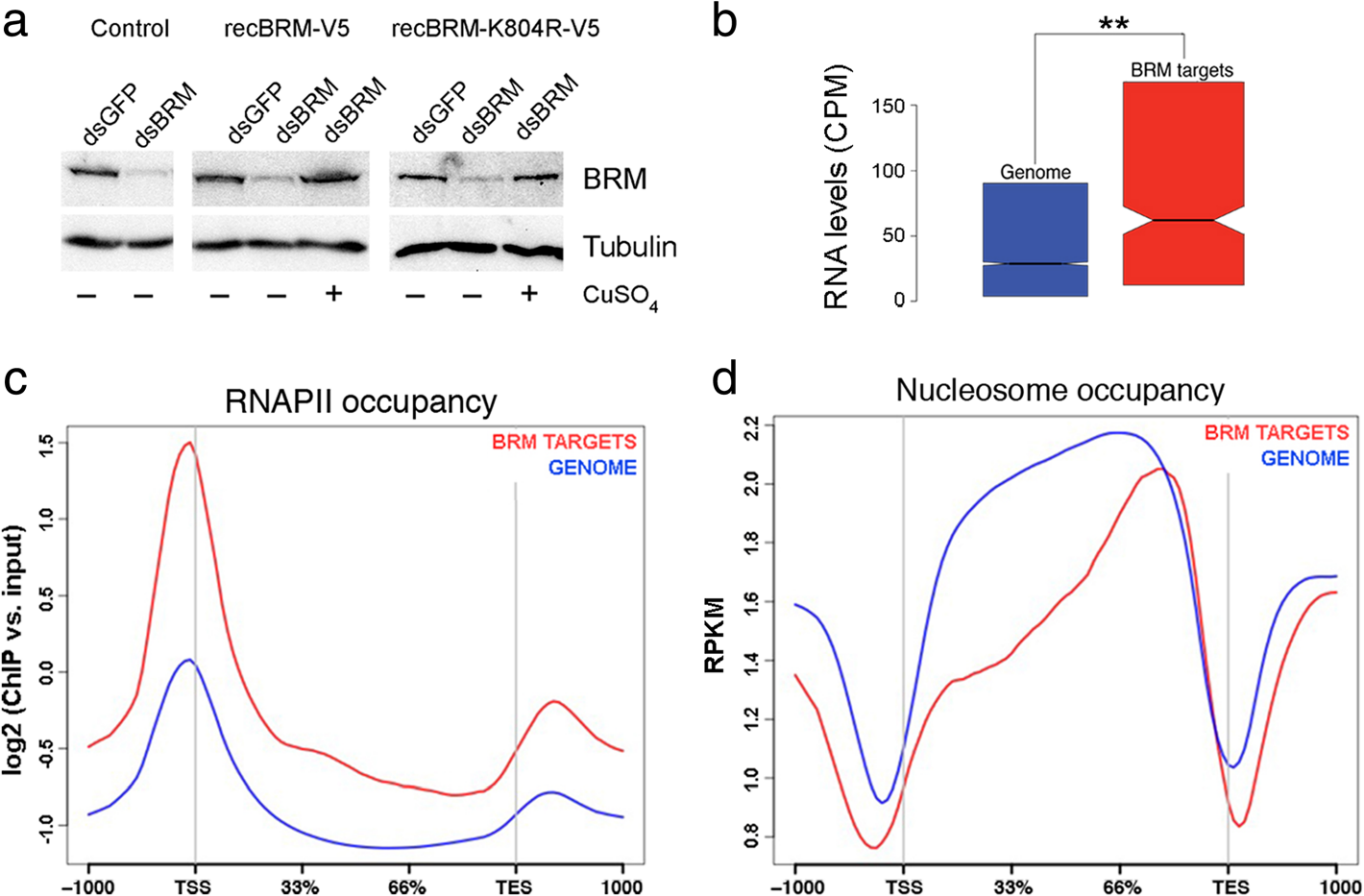
The confident BRM target genes in S2 cells. Control cells and cells stably transfected with expression vectors for recBRM-V5 and recBRM-K804R-V5 were treated with dsRNA to knock down BRM. Control cells were treated in parallel with dsRNA complementary to GFP. The expression of the recombinant proteins was induced with CuSO_4_, as indicated. **a** Western blot analysis of BRM levels using an antibody against the endogenous BRM. Tubulin was used as a loading control. **b** Box plot showing the average expression levels of the BRM target genes compared with the average expression level of all the genes in S2 cells. **c**-**d** Metagene analyses of RNAPII and nucleosome occupancy in the BRM target genes (red, *n* = 541) and flanking sequences (X-axis) compared with the average distributions in all the S2 genes (blue, n = 13,294 genes)

We were interested in identifying the genes that are regulated by BRM and in distinguishing ATPase-dependent from ATPase-independent regulatory events. To this aim, we chose to follow a mild overexpression approach. The levels of BRM expression are lower in S2 cells than in many other fly tissues in vivo (modENCODE data available at FlyBase http://flybase.org/reports/FBgn0000212.html), and we argued that a slight overexpression in S2 cells would mimic the natural levels of BRM expression observed in tissues where SWI/SNF plays relevant physiological roles. We depleted the endogenous BRM by RNA interference (RNAi) in S2 cells, and we slightly overexpressed V5-tagged recombinant BRM proteins, either wild-type BRM (recBRM-V5) or a catalytically inactive BRM mutant (recBRM-K804R-V5) (Fig. 2a). We then carried out RNA-seq analyses to describe the effects of the treatments on the S2 transcriptome.

We first analyzed the transcriptomes of the S2 cells that slightly overexpressed wild-type BRM in a depletion background, and identified 2112 genes with differential expression compared to control cells. Out of the 2112 genes, 541 were BRM-bound (245 decreased and 296 increased expression levels). This group of 541 genes occupied by BRM and affected by expression of wild-type BRM constituted a confident set of BRM target genes in S2 cells (Additional file 1: Table S1 and Additional file 1: Figure S2) and were studied further.

A gene ontology (GO) classification of biological processes showed a large functional diversity among the BRM target genes, many of which encode regulatory proteins and enzymes, and supported the involvement of BRM in important biological processes such as metabolism, cell communication, immune response and development (Additional file 1: Figure S3). The BRM target genes were more expressed in S2 cells than the rest of the genes (Fig. 2b), and were occupied by BRM in a bimodal fashion, with highest occupancy upstream of the TSS, as for the total of the BRM bound genes (compare Fig. 1a and Additional file 1). Moreover, the genes repressed by BRM in S2 cells were characterized by higher levels of BRM in the gene bodies than the upregulated genes (Additional file 1: Figure S4).

The BRM targets were also characterized by higher RNAPII levels (Fig. 2c) and lower nucleosome levels (Fig. 2d) than the average of the genome, in both the gene body and flanking regions. Interestingly, 46% of the identified targets (245 out of 541) were repressed by BRM, which points to a dual active/repressive role for BRM in transcription regulation and shows that the repressive function of BRM is very prevalent in the fly genome. The genes that were repressed by BRM showed on average higher RNAPII occupancy and lower nucleosome density in the gene body than the genes upregulated by BRM (Additional file 1: Figure S5).

### The genes regulated by BRM through ATPase-independent mechanisms

**Fig. 3:**
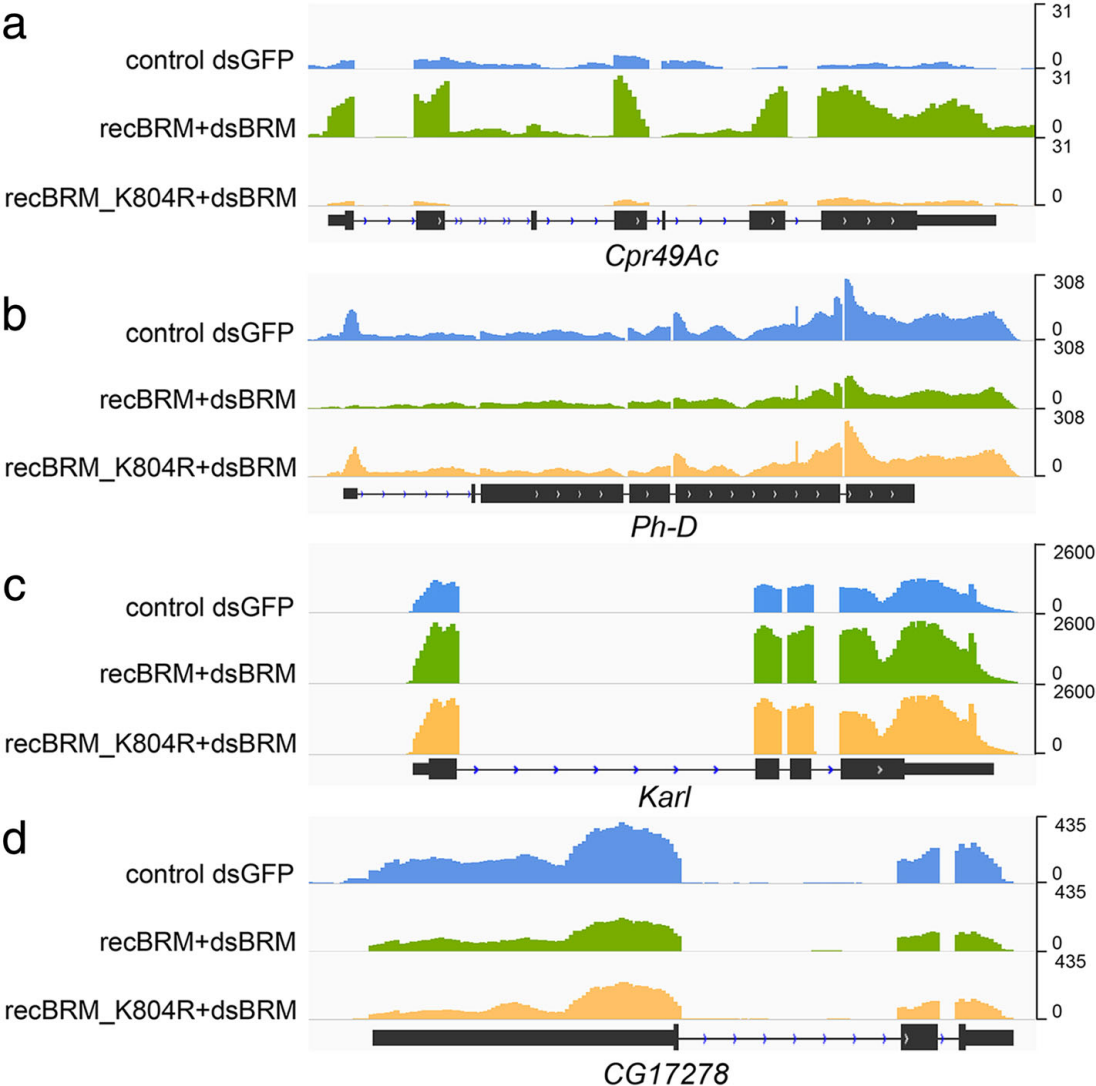
Examples of the effect of BRM expression on transcript levels. RNA-seq of total RNA purified from control S2 cells and from cells that expressed recombinant BRM, either recBRM-V5 or recBRM-K804R-V5, in a depletion background. **a**-**b** Examples of ATPase-dependent genes. The RNA levels of *Cpr49Ac* and *Ph-D* are increased and decreased, respectively, by recBRM-V5 expression but not by expression of the catalytically inactive recBRM-K804R-V5. **c**-**d** Two examples of ATPase-independent genes. Expression of either recBRM-V5 or recBRM-K804R-V5 have similar effects on the RNA levels of *Karl* and *CG17278*

We then compared the effect of expressing wild-type BRM with that of expressing the ATPase mutant on the transcriptome of S2 cells. The rationale of the experiment was that genes regulated by BRM in an ATPase-independent manner (called “ATPase independent genes” from here on) would be similarly affected by expression of either active or inactive BRM. Of the 541 genes that were affected by the expression of wild-type BRM, 270 were also affected by expression of the mutant protein, and the change was in the same direction (either increased or decreases in both treatments) in 267 out of these 270 cases. We concluded that this group of 267 genes (49.35% of target genes) are regulated through mechanisms that involve BRM but do not require its catalytic activity (Fig. 3). Therefore, such mechanisms must be different from conventional ATP-dependent chromatin remodeling by SWI/SNF.

We considered the possibility that the effects observed in cells that expressed the ATPase mutant could be due to the low levels of endogenous active BRM, not to the function of the mutant protein. If so, BRM depletion should reproduce the effect of expressing mutant BRM, which was not the case. Only 24 out of the 267 ATPase independent genes were differentially expressed in BRM-depleted cells compared to control dsGFP cells (Additional file 1: Table S2). This observation rules out the possibility that the observed effects are due to low levels of active BRM.

Of the 541 BRM targets identified above, 274 reacted differently to the expression of wild-type and mutant BRM. The mutant protein lacks ATPase activity, but is able to assemble into SWI/SNF complexes (reference [20] and Additional file 1: Figure S6). Moreover, ChIP-seq analysis of recombinant BRM proteins showed that both the wild-type and the mutant BRM were recruited to the genomic targets identified above (Additional file 1: Figure S7). We also analyzed the association of wild-type and mutant BRM with the ATPase-dependent and ATPase-independent genes using metagene profile comparisons and correlation analyses (Additional file 1: Figure S8). These comparisons supported the conclusion that the wild-type and the non-catalytic mutant BRM are recruited to both ATPase-dependent and ATPase-independent genes, which rules out differences due to defects in SWI/SNF targeting. Thus, we concluded that the 274 genes that reacted differently to the expression of wild-type and mutant BRM are regulated in an ATPase-dependent manner (Additional file 1: Table S1, Figure 3).

The levels of recombinant BRM, either wild-type or mutant, were higher in the gene body and lower downstream of the TES in the ATPase-dependent genes than in the ATPase-independent ones, as observed for endogenous BRM (compare the metagene plot in Fig. 5b with the plots in Additional file 1: Figure S8A). The relative abundance of the recombinant BRM proteins at the TSS was instead different from that of endogenous BRM. The fact that different antibodies were used to immunoprecipitate the recombinant and endogenous proteins could explain this difference.

**Fig. 4:**
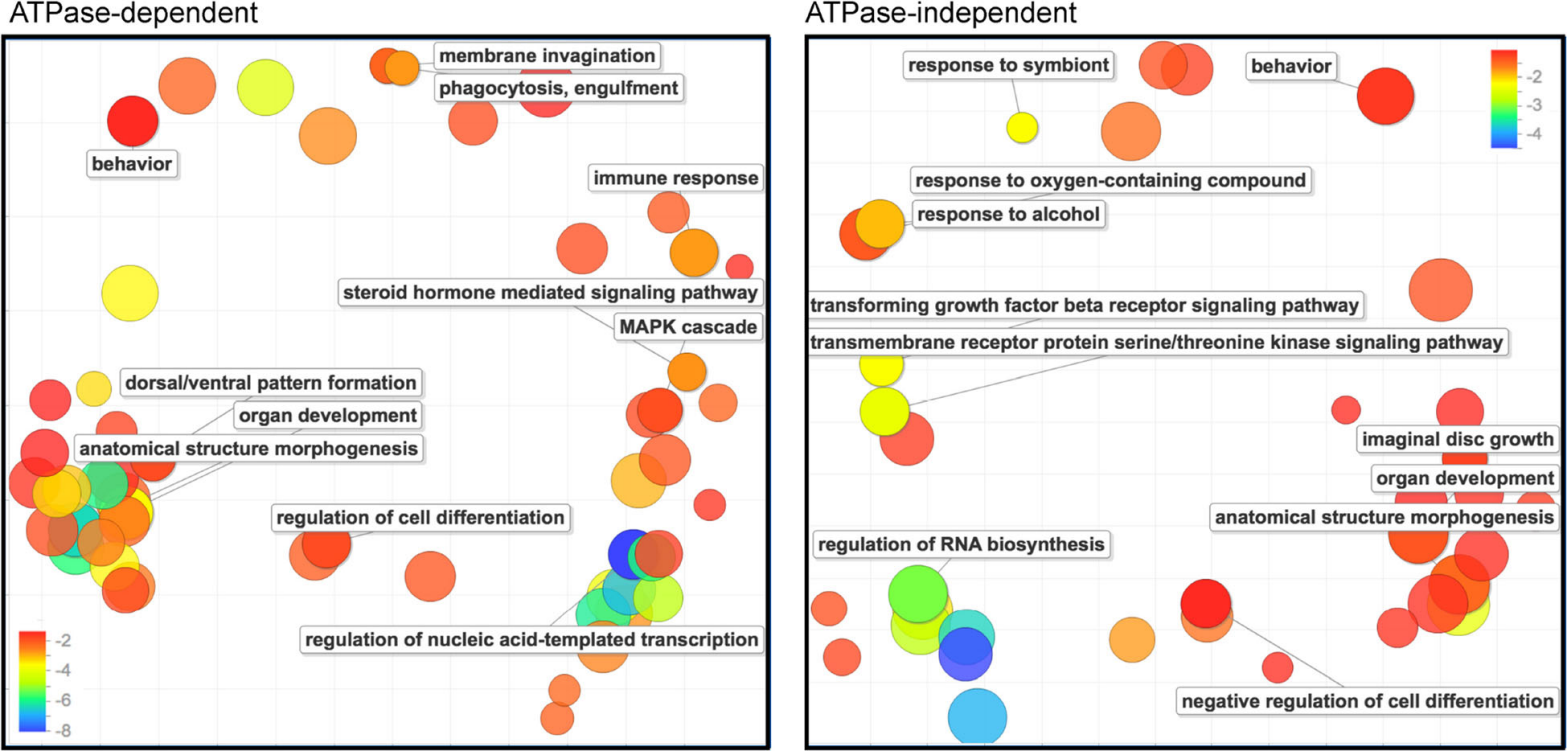
The biological functions of ATPase-dependent and ATPase-independent genes. Gene ontology enrichment tests were carried out for the ATPase-dependent and ATPase-independent genes using GOrilla. The scatterplots show gene ontology cluster representatives for the ATPase-dependent and ATPase-independent genes that showed decreased expression in response to BRM expression. The two-dimensional clustering was based on semantic similarity. Bubble color indicates the corrected probability value for the enrichment, in a log10 scale. Probability values were corrected for multiple testing. Bubble size indicates the frequency of the GO term in the underlying GO database (bubbles of more general terms are larger). The plots were generated by REVIGO based on the lists of enriched GO terms for genes that showed decreased expression in response to BRM expression. The genes upregulated by BRM expression did not show any significant enrichment

We carried out GO enrichment tests with the ATPase-dependent and ATPase-independent targets. Both groups of genes were significantly enriched in GO terms related to regulation of cell cycle and development, metabolism, signal transduction and transcription (Fig. 4). The group of ATPase-dependent genes was also significantly enriched in genes related to membrane invagination, phagocytosis and engulfment, processes that were not represented among the ATPase-independent genes. Interestingly, different signaling pathways were linked to the ATPase-dependent and ATPase-independent groups. Steroid hormone-mediated signaling and the MAPK pathway, in particular the JNK cascade, were highly enriched in the ATPase-dependent genes, whereas enzyme-linked receptor signaling, for example the growth factor-beta (TGFB) receptor pathway, was significantly represented in the ATPase-independent group. In summary, the ATPase-dependent and ATPase-independent genes have specialized roles in the regulation of specific cellular and developmental processes.

### The promoters of the BRM target genes

**Fig. 5:**
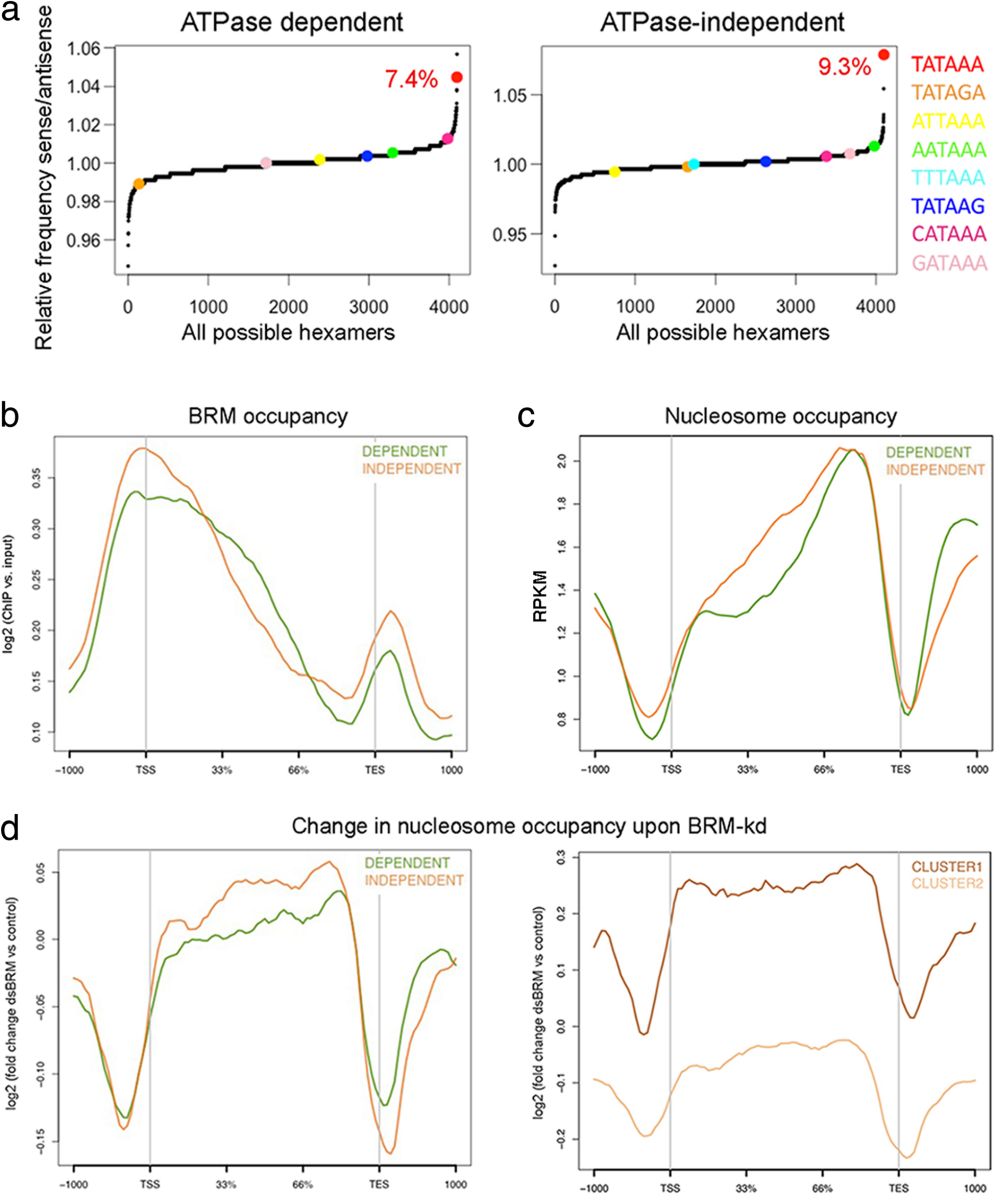
Characterization of the ATPase-dependent and ATPase-independent genes. **a** Analysis of TATA-box occurrence. The frequencies of all possible hexamers in the regions upstream (− 50/− 10 bp) of the TSS were calculated for BRM target genes. The plots show the log2 ratio of total occurrence number on the sense versus the antisense strand of the canonical TATAAA sequence and of alternative TATA box sequences in the two groups of genes. The red figures give the percentages of genes with the canonical TATAAA box. **b**-**c** Metagene analyses of BRM and nucleosome occupancy in the ATPase-dependent (green, *n* = 274 genes) and ATPase independent genes (brown, *n* = 267 genes). The distribution of BRM around the TSS is significantly different in the two groups (K-S test, *p* = 0.0001). The average nucleosome density in the gene body is significantly higher in the ATPase-independent genes (K-S test, *p* = 0.009). **d** Metagene analysis of changed nucleosome occupancy upon BRM depletion (K-S test, *p* = 0.002). The right panel shows the metagene of changed nucleosome occupancy for Clusters 1 (dark brown, *n* = 62 genes) and 2 (light brown, *n* = 205 genes), two groups of ATPase-independent genes defined by changes in nucleosome density upon BRM depletion. Cluster 1 shows a very high increase of nucleosome occupancy in cells depleted of BRM

Only 7.4 and 9.3% of the promoters of the ATPase-dependent and ATPase-independent genes, respectively, had consensus TATA boxes, which was significantly lower than the genome average (19% average genome according to reference [26], *p* < 0.01), particularly for the genes that were downregulated by BRM (Fig. 5a and Additional file 1: Figure S9). We concluded that most BRM target genes have TATA-less promoters.

The promoters of the BRM targets were highly enriched in binding motifs for different transcription factors, mostly homeobox proteins. Some of the enriched motifs were common for ATPase-dependent and ATPase-independent genes, but many others were specific (Additional file 1: Table S3). The enrichment of certain DNA-binding motifs in the promoters of the BRM targets could be due to secondary events caused by changes in the level of expression of the identified transcription factors. However, the expression of the majority of the identified transcription factors was not changed in cells that expressed wild-type or mutant BRM (Additional file 1: Table S4), which supports the conclusion that the observed effects are to a large extent direct. In summary, the motif enrichment analysis suggests that the ATPase-dependent and ATPase-independent genes are characterized by different promoter configurations. The abundance of motifs for developmental regulators in both the ATPase-dependent and ATPase-independent genes is in agreement with the GO analysis reported above and confirms that both groups of genes play roles in the regulation of developmental processes.

### Differential features of genes regulated by BRM through ATPase-dependent and ATPase-independent mechanisms

The average gene length was similar in the ATPase-dependent and ATPase-independent genes (8.3 and 9 kB, respectively, *p* = 0.76). Instead the average expression level was slightly lower for the ATPase-independent genes (average CPM values were 236 vs 127; *p* = 0.048). The ATPase-independent group was also characterized by higher BRM binding levels at the TSS (Fig. 5b) and higher nucleosome occupancy in the gene body than the ATPase-dependent genes (Fig. 5c). The binding and distribution of RNAPII were very similar in both groups (Additional file 1: Figure S10).

Analysis of MNase data from Shi et al. [25] revealed that BRM depletion causes a remarkable drop of nucleosome occupancy upstream of TSSs and downstream of TESs, for both groups of genes (Fig. 5d), which reveals an important role for SWI/SNF in maintaining the nucleosome configuration in genomic regions that flank transcription units. Moreover, BRM depletion caused a significant increase in nucleosome occupancy in the gene bodies of the ATPase-independent genes, which implies that a major function of BRM in this group of genes is to keep nucleosome density low through non-catalytic mechanisms. On the other hand, BRM depletion did not cause any major change in nucleosomes occupancy in the gene bodies of the ATPase-dependent genes (Fig. 5d). This difference reveals a dual function of BRM in the regulation of ATPase-dependent and ATPase-independent genes.

Next, we performed clustering analysis on the ATPase-independent genes and identified a subset of 62 genes for which the average increase in nucleosome density upon BRM depletion was most pronounced (Cluster 1 in Fig. 5d, right panel). Interestingly, the genes in Cluster 1 were on average twice as long as the average gene in the fly genome (8.4 kb compared to 4.75, *p* = 0.01). Taken together, these observations suggest that BRM acts to reduce nucleosome density in the gene bodies of these long genes, probably to regulate transcription elongation, and that it does so through an ATPase-independent mechanism. Cluster 1 is significantly enriched in lipid-binding proteins (GO:0008289, FDR q-value = 0.01) and includes genes that code for proteins with important roles in signal transduction and juvenile hormone function, which strengthens the conclusion that the ATPase-independent genes mediate important biological roles.

## Discussion

The most striking observation derived from our present study in S2 cells is the high number of genes that are regulated by SWI/SNF through non-catalytic mechanisms. S2 cells cannot fully recapitulate the functions of SWI/SNF in fly development and physiology, but results from previous differential gene expression studies in flies are compatible with the broad incidence of the non-catalytic function of BRM also in vivo. In a study by Zraly et al. [22], only a fraction of the genes that were deregulated in flies expressing a temperature-sensitive snr1 mutant were also significantly affected in flies that expressed the catalytically inactive *Brm*^*K804R*^ mutant. A possible interpretation of these results is that a fraction of SWI/SNF-dependent genes does not require catalytically active BRM and is therefore normally expressed in the presence of *Brm*^*K804R*^*.* This observation suggests that the ATPase-independent function of BRM is widespread in vivo.

Single-gene studies have provided clues about the nature of the mechanisms by which SWI/SNF regulates transcription without the need of ATP-driven nucleosome remodeling by BRM. For example, SWI/SNF controls the timing of *ftz-f1* transcription during metamorphosis in *D. melanogaster* by physically obstructing transcription elongation [27]*.* In the absence of ecdysone, SWI/SNF occupies a region located in the *ftz-f1* gene body and acts as a chromatin barrier that pauses RNAPII elongation. The presence of BRM in gene bodies revealed by our ChIP-seq study suggests that this mechanism could be common in S2 cells. On the other hand, SWI/SNF can act by recruiting transcription regulators [7, 28, 29]. For example, in the control of circadian rhythms in *D. melanogaster*, BRM represses the expression of the circadian genes *per* and *tim* by recruiting repressors to the promoters of these genes, and this recruitment does not require BRM’s ATPase activity [23]. At the same time, BRM increases nucleosome density in these genes through ATPase-dependent chromatin remodeling to avoid excessive deposition of repressive marks [23]. SWI/SNF is known to interact with histone deacetylases and co-repressors, including Sin3A-HDAC, NCoR and Polycomb [30–32], and also with positive regulators of transcription such as H3K27 demethylases, the trithorax-group protein zeste, and the Mediator [28, 33, 34]. Because of its extensive interaction network, SWI/SNF has the potential to act as a platform for protein-protein interactions and, in an ATPase-independent manner, recruit other chromatin regulators to activate or repress the expression of many genes. In some genes, the catalytic and non-catalytic activities of BRM may collaborate as they do in the regulation of *per* and *tim* [23]. In many other cases, as shown by our results, the catalytic activity of BRM is dispensable.

## Conclusions

The function of SWI/SNF in transcription regulation has long been attributed to the ability of this complex to remodel nucleosomes, which requires ATP hydrolysis by BRM. Here we have identified hundreds of genes in the genome of *Drosophila melanogaster* that are regulated by SWI/SNF through mechanisms that do not require BRM to have an ATPase activity. Therefore, such mechanisms must be different from conventional ATP-dependent chromatin remodeling by SWI/SNF. Moreover, the ATPase-dependent and ATPase-independent mechanisms of transcription regulation by SWI/SNF operate on different sets of genes that have different promoter configurations and are linked to different biological functions, which reveals a novel level of specialization in the mechanisms of SWI/SNF action.

## Methods

### Cell culture

*Drosophila melanogaster* S2 cells were purchased from Invitrogen and cultured in Schneider’s *Drosophila* medium (Invitrogen) containing 10% heat-inactivated FBS, 50 U/ml penicillin and 50 μg/ml streptomycin at 28 °C.

### Antibodies

The anti-BRM antibody used for Western blotting was raised in rabbit and has been characterized in a previous study [13]. An antibody raised against the rat BRG1 protein by Östlund-Farrants et al. [35] was used to detect BRM in S2 cells. The specificity of this antibody in *D. melanogaster* has been previously documented [13]. The antibodies against MOR and SNR1 were kindly provided by C. P. Verrijzer (see reference [36] and references therein). The monoclonal anti-alpha-tubulin antibody (clone B-5-1-2) was from Sigma-Aldrich and the anti-V5 antibodies were from Invitrogen (R96025) or Abcam (ab9116).

### RNA interference in S2 cells

RNAi experiments in S2 cells were carried out as previously described by Tyagi et al. [13]. Double-stranded RNAs complementary to BRM or GFP were synthesized by in vitro transcription using the MegaScript RNAi kit (Ambion) from gene-specific PCR fragments with incorporated T7 promoters at both ends. The sequences of the PCR primers used for dsRNA synthesis are provided in Table S5 (Additional file 1). 3 × 10^6^ S2 cells were cultured in 6-well plates overnight, washed with serum-free and antibiotic-free Schneider’s medium, and treated with 30 μg of dsRNA/well. The cells were harvested 48 h after the addition of dsRNA.

### Expression of recombinant BRM proteins in S2 cells

The stably transfected cell lines for expression of recBRM-V5 and recBRM-K804R-V5 were constructed and cultivated as described in Yu et al. [15]. The expressions of recBRM-V5 and recBRM-K804R-V5 were under the control of the metallothionein promoter of the pMT vector (Invitrogen), which can be modulated using different concentrations of copper sulfate. For RNA-seq and ChIP-seq experiments, the expression of the endogenous BRM in the stably transfected cells was knocked down by RNAi, and the recombinant BRM proteins were expressed. To this end, the cells were first treated with dsRNA as indicated above, 100 μM copper sulfate was added to the cultures 24 h after the start of the RNAi treatment, and the incubation was allowed to proceed for additional 24 h.

### SDS-PAGE and western blotting

Cell pellets were resuspended in SDS-PAGE sample buffer supplemented with 8 M urea and heated to 100 °C to denature and extract proteins. Proteins were separated by SDS-PAGE and transferred to polyvinylidenefluoride membrane in Tris-glycine buffer with 0.02% SDS and 4 M urea using a semi-dry electrophoretic transfer cell. The antibodies were diluted in PBS containing 0.05% Tween-20 and 1% low-fat powder milk, and antibody incubations were carried out following standard procedures. The ECL system (GE Healthcare) was used for the chemiluminiscent detection of secondary antibodies conjugated to horseradish peroxidase.

### Co-immunoprecipitation

S2 cells were harvested and resuspended in PBS supplemented with 0.2% NP40, and homogenized using a glass homogenizer with a tight-fitting pestle. The homogenate was centrifuged at 1.500 *g* for 10 min at 4 °C. The pellet containing the nuclei was resuspended in PBS, sonicated and centrifuged at 16.300 *g* for 10 min at 4 °C. The resulting supernatant was supplemented with 0.1% NP40, cleared by centrifugation at 13.200 rpm for 15 min and used as input for immunoprecipitation reactions following standard procedures. Antibody incubations were overnight under rotation at 4 °C. Samples were washed in PBS supplemented with 0.1% NP40 for four times 5 min. The bound proteins were eluted with 1% SDS, precipitated with acetone and analyzed by SDS-PAGE and Western blotting.

### ChIP-seq

Chromatin was extracted from S2 cells as previously described by Hessle et al. [37]. In short, the fixed cells were lysed and sonicated, the lysates were cleared by centrifugation, and antibodies against either endogenous BRM or V5 (Invitrogen) were added and incubated overnight under rotation. Protein-G coated magnetic beads were added to the samples and the incubation was allowed to proceed for one additional hour. The beads were then washed in 0.8% RIPA buffer for four times for 10 min each. The beads were resuspended in TE buffer containing 0.5% SDS, 0.1 mg/ml RNase A and 0.2 mg/ml proteinase K, and incubated at 55 °C for 3 h and at 65 °C overnight to reverse the crosslinking. The immunoprecipitated DNA was extracted with a mixture of phenol, chloroform and isoamylalcohol (Sigma). For qPCR analyses, the KAPA SYBR Fast qPCR Kit (KAPA Biosystem) was used in a QIAGEN Rotor-Gene Q system. The amplification efficiency for each primer was measured in each qPCR run. Standard curves for each primer-pair were used to quantify the immunoprecipitated DNA. The ChIP-qPCR signals were normalized using a DIG-DNA-antibody complex [38]. For ChIP-seq, 10 ng of both input DNA and immunoprecipitated DNA were used for Rubicon Thruplex-FD library preparation and sequenced in an Illumina HiSeq2500 instrument to an average depth of 39.6 million single-end 50 bp-reads per sample. Library construction and sequencing were carried out at the Science for Life Laboratory (Stockholm, Sweden). High-quality sequencing tags, as determined by inspection with FastQC, were aligned to the *Drosophila melanogaster* genome using build BDGP6 with Bowtie 2 [39] allowing for up to two mismatches. The alignment files were indexed using SAMtools [40]. For metagene and heatmap plotting purposes the bam files for two BRM-IP biological replicates were merged into one file and compared to a merge of the two biological replicates of their corresponding Inputs. The ngs.plot package [41] was used to calculate BRM enrichment levels throughout the genome as well as for metagene and heatmap representations. Statistical significance between different metagene distributions was calculated using the Kolmogorov-Smirnov (K-S) test in GraphPad. A fragment size of 200 bp was selected as input parameter to calculate the read coverage, based on the average sonicated DNA fragment size we observed in our Bioanalyzer runs. Average values of BRM enrichment over the genebody region and up to 200 bp upstream of the TSS and downstream of the pA site were calculated. Genes were considered as bound by BRM if the average values were ≥ 1.1 (FC IP/Input). We also used MACS2 [42] to call peaks of BRM binding. Duplicated reads were first removed from the alignment files with the filterdup function, and peaks were called using the IP alignment as Treatment and the Input as the Control with the following settings: bandwidth = 300, pvalue = 0.01, extension size of the fragments = 200, broad region calling = on, cutoff for broad region = 0.1.

### RNA-seq

Control S2 cells and S2 cells stably transfected with expression plasmids for either recBRM-V5 or recBRM-K804R-V5 were treated with BRM-dsRNA for 48 h to deplete endogenous BRM. The same cell lines were treated in parallel with GFP-dsRNA as a control. Total RNA was isolated using the RNAqueous kit (Ambion) and used to construct poly(A) libraries for massive parallel sequencing on an Illumina HiSeq2000 sequencer, high-output mode, to a depth of 10–15 M paired-end reads per sample (2 × 125 bp). Library construction and sequencing were carried out at the Science for Life Laboratory (Stockholm, Sweden) using NEBNext Ultra Directional RNA library prep kit for Illumina with 1 microgram total RNA as starting material. Two biological replicates were sequenced independently for each cell line. Illumina fastq files were inspected with FastQC to assess the quality of the paired-end reads. RSeQC 2.3.6 [43] and Preseq 2.0 [44] packages were used to check that mapping statistics, read distribution over structural parts of genes, uniformity of gene body coverage, strandedness, and library complexity were all in line with a typical strand-specific RNA-seq experiment. High-quality reads were mapped with TopHat2 [45] to the *Drosophila melanogaster* genome assembly, build BDGP6 (Dm3). BAM alignments were indexed with SAMtools [40]. Alignments were converted into bigWig tracks with the bamCoverage function implemented in DeepTools [46] and used for visual inspection with the IGV genome browser [47]. Read counts and FPKMs were generated using HTSeq [48] and Cufflinks tools [49], respectively. The Limma package [50] was used for differential gene expression analysis using raw reads from the independent replicates as input. Since correlation analysis of biological replicates showed high reproducibility (Additional file 1: Figure S11), the reads from both experiments were merged and indexed with SAMtools [40] and analyzed together for metagene and heatmap representations.

### Promoter sequence analysis

Gene sequences extending from nucleotide − 50 to − 10 bp upstream of the TSSs of the genes of interest were retrieved using the Regulatory Sequence Analysis Tools (http://rsat.sb-roscoff.fr/). Alternative TSSs were also included. We then used an unbiased exhaustive enumeration method to look for hexamers that were more enriched in the sense strand than in the antisense strand [51], using the Biostrings and Stringr packages in the software package R. The total occurrence number of each hexamer was calculated on both sense and antisense strand. The log2 ratio of the total occurrence number on the sense and antisense strands was then calculated and plotted. Sequences between − 300 bp upstream of the TSS and + 100 bp downstream of the TSS were retrieved and used for motif enrichment analysis using CentriMo (http://meme-suite.org/tools/centrimo).

### BRM, nucleosome and RNAPII occupancy analysis

ChIP-seq data for endogenous BRM is available in the Gene Expression Omnibus repository with accession number GSE95236). GFP_control and dsBRM MNase-seq datasets from Shi et al. [25] were downloaded from ArrayExpress (E-MTAB-1967). RNAPII ChIP and Input files from Lam et al. [52] were downloaded from ArrayExpress (E-MTAB-1084). The antibody used in the RNAPII ChIP-seq experiment was directed against Rpb3 [52]. All sequencing files were processed as described above for our ChIP-seq datasets. Normalized average density plots around the TSS were drawn with the ngs.plot software [41]. The statistical robustness parameter, which filters out 0.5% of genes with the most extreme expression values, was applied to all calculations.

### Gene ontology

GO classification was done using the PANTHER Classification System [53]. GO enrichment tests were carried out with GOrilla [54] using the 9872 genes detected in the RNA-seq analysis as background list. Probability values were corrected for multiple testing and the significance cutoff was *p* = 0.05. GO terms that were significantly enriched in the GOrilla nalaysis were summarized and visualized using Revigo [55].

## Additional file

Additional file 1: **Figure S1.** Comparison of input-corrected enrichment values for RNAPII and BRM. **Table S1.** List of BRM target genes in S2 cells. **Figure S2.** Heat map of normalized gene expression of BRM target genes. **Figure S3.** Gene ontology classification of BRM target genes. **Figure S4.** Metagene analysis of BRM in the BRM target genes. **Figure S5.** Metagene analysis of RNAPII and nucleosome occupancies in BRM target genes that are upregulated or downregulated by BRM. **Table S2.** List of genes differentially expressed in BRM-depleted cells. **Figure S6.** recBRM-V5 and recBRM-K804R-V5 are incorporated into SWI/SNF complexes. **Figure S7.** Genomic distribution of recBRM-V5 and recBRM-K804R-V5 in S2 cells analyzed by ChIP-seq. **Figure S8.** Distributions of recBRM-V5 and recBRM-K804R-V5 in ATPase dependent and ATPase-independent genes. **Figure S9.** Analysis of TATA-box occurrence in the promoters of the BRM target genes. **Table S3.** List of enriched motifs in the promoters of BRM target genes. **Table S4.** Average fpmk values for selected transcription factors. **Figure S10.** Metagene analysis of RNA polymerase II distribution in the ATPase-dependent and ATPase-independent genes. **Table S5.** Sequences of PCR primers used for dsRNA synthesis. **Figure S11.** RNA-seq experiments: correlation analysis of biological replicates. (PDF 4624 kb)

## Abbreviations

BRM: Brahma
BRG1: Brahma-related gene 1
ChIP: Chromatin immunoprecipitation
GO: Gene ontology
MNase: Micrococcal nuclease
qPCR: Quantitative PCR
RNAi: RNA interference
RNAPII: RNA polymerase II
TES: Transcription end site
TSS: Transcription start site
