# Supplementary material for: SWI/SNF regulates half of its targets without the need of ATP-driven nucleosome remodeling by Brahma
Source: BMC Genomics. 2018 May 18;19:367. doi: 10.1186/s12864-018-4746-2 (PMC5960078; doi:10.1186/s12864-018-4746-2)
Supplement: Supplementary file 1 — Figure S1. Comparison of input-corrected enrichment values for RNAPII and BRM. Table S1. List of BRM target genes in S2 cells. Figure S2. Heat map of normalized gene expression of BRM target genes. Figure S3. Gene ontology classification of BRM target genes. Figure S4. Metagene analysis of BRM in the BRM target genes. Figure S5. Metagene analysis of RNAPII and nucleosome occupancies in BRM target genes that are upregulated or downregulated by BRM. Table S2. List of genes differentially expressed in BRM-depleted cells. Figure S6. recBRM-V5 and recBRM-K804R-V5 are incorporated into SWI/SNF complexes. Figure S7. Genomic distribution of recBRM-V5 and recBRM-K804R-V5 in S2 cells analyzed by ChIP-seq. Figure S8. Distributions of recBRM-V5 and recBRM-K804R-V5 in ATPase dependent and ATPase-independent genes. Figure S9. Analysis of TATA-box occurrence in the promoters of the BRM target genes. Table S3. List of enriched motifs in the promoters of BRM target genes. Table S4. Average fpmk values for selected transcription factors. Figure S10. Metagene analysis of RNA polymerase II distribution in the ATPase-dependent and ATPase-independent genes. Table S5. Sequences of PCR primers used for dsRNA synthesis. Figure S11. RNA-seq experiments: correlation analysis of biological replicates. (PDF 4624 kb) [file 12864_2018_4746_MOESM1_ESM.pdf]

SWI/SNF regulates half of its targets without the need of ATP-driven nucleosome remodeling by Brahma

Antonio Jordán-Pla, Simei Yu, Johan Waldholm, Thomas Källman, Ann-Kristin Östlund Farrants and Neus Visa

Additional file 1

Figure S1. Comparison of input-corrected enrichment values for RNAPII and BRM

Table S1. List of BRM target genes in S2 cells

Figure S2. Heat map of normalized gene expression of BRM target genes

Figure S3. Gene ontology classification of BRM target genes

Figure S4. Metagene analysis of BRM in the BRM target genes

Figure S5. Metagene analysis of RNAPII and nucleosome occupancies in BRM target genes that are upregulated or downregulated by BRM

Table S2. List of genes differentially expressed in BRM-depleted cells

Figure S6. recBRM-V5 and recBRM-K804R-V5 are incorporated into SWI/SNF complexes

Figure S7. Genomic distribution of recBRM-V5 and recBRM-K804R-V5 in S2 cells analyzed by ChIP-seq

Figure S8. Distributions of recBRM-V5 and recBRM-K804R-V5 in ATPase dependent and ATPase-independent genes

Figure S9. Analysis of TATA-box occurrence in the promoters of the BRM target genes

Table S3. List of enriched motifs in the promoters of BRM target genes

Table S4. Average fpmk values for selected transcription factors

Figure S10. Metagene analysis of RNA polymerase II distribution in the ATPase-dependent and ATPase-independent genes

Table S5. Sequences of PCR primers used for dsRNA synthesis

Figure S11. RNA-seq experiments: correlation analysis of biological replicates

Table S1. List of BRM responsive genes in S2 cells

Seven genes directly upregulated by copper sulfate were excluded from the analysis **(**FBgn0011672, FBgn0024236, FBgn0030343, FBgn0035432, FBgn0039714, FBgn0062412, FBgn0062413**)**

ATPase-dependent increased (132 genes)

alt	CG15279	CG4500	GstD10	nord
AnnIX	CG16718	CG5399	GstD2	Npc1b
Aplip1	CG17292	CG7149	GstS1	Ntf-2
Arc1	CG17574	CG7362	Hr4	Oseg5
capt	CG18467	CG7408	Hsp26	Pfk
CG10337	CG18812	CG7702	Hsp67Bc	pgant4
CG10550	CG2993	CG7737	Hsp70Aa	PGRP-SD
CG10559	CG30197	CG8026	Hsp70Ab	Pros28.1
CG10657	CG3078	CG8501	htl	Psa
CG10869	CG31053	CG9168	Ilp6	Rcd2
CG11313	CG31370	CG9171	ImpL3	sar1
CG11395	CG31522	CG9815	Jheh1	Sema-1b
CG11608	CG31688	CheB42c	Keap1	Slc45-1
CG11637	CG31798	chic	l(2)k01209	SMSr
CG11786	CG31955	chrb	l(2)s5379	Spn31A
CG12643	CG31974	Cht4	Lapsyn	TER94
CG12693	CG32373	cib	ltd	Tk
CG13004	CG32407	Cpr	m	Tpc1
CG13575	CG3280	Cpr49Ac	mbc	Trx-2
CG13791	CG33129	CycG	MFS3	tsh
CG13893	CG33136	Cyp6a8	miple	TwdlF
CG14207	CG34263	Cyp9f2	Muc30E	Ugt58Fa
CG14274	CG41087	debcl	Myo31DF	vsg
CG14855	CG41284	Dhap-at	nes	wun
CG14995	CG42402	Dro	nimB1	
CG15048	CG43886	G-ialpha65A	nimB2	
CG15082	CG4461	glob3	nimB5	

ATPase-dependent decreased (139 genes)

ade5	CG12446	CG14410	CG32164	CG42666
Adf1	CG12576	CG14419	CG32425	CG42668
ap	CG12768	CG15161	CG32626	CG42669
bab2	CG13001	CG15820	CG33298	CG43246
bowl	CG13116	CG1620	CG34449	CG43248
Btk29A	CG13510	CG18622	CG34459	CG4496
CG10011	CG13624	CG30424	CG3764	CG4502
CG11357	CG13836	CG31211	CG3838	CG4753
CG1146	CG13868	CG32103	CG4061	CG5773
CG11486	CG1427	CG32138	CG42240	

CG6966	Dif	Ir	nau	rpr
CG7231	dl	JIL-1	nej	sbb
CG7781	DOR	Kdm2	NK7.1	Set2
CG8034	dos	kdn	Npc2b	Sik3
CG8173	Drak	ken	Oatp74D	skd
CG8369	EcR	kibra	Or19b	slik
CG9005	Eip75B	kuk	pcs	SoxN
CG9119	fl(2)d	L	Pdcd4	spg
CG9674	ftz-f1	l(3)L1231	ph-d	Swim
CG9837	GATAe	lbk	pnt	Tak1
chinmo	gol	lola	psq	tna
crp	gpp	mab-21	Pvr	tou
Csk	HDAC4	mael	px	Ubc-E2H
csw	hdc	Mekk1	rdgB	upd2
cv-c	hep	Mes2	Reck	vri
cwo	Idgf2	meso18E	RhoGAP15B	whd
Cyp18a1	inx2	MESR3	RhoGAP18B	zfh2
Cyp6t1	IP3K1	msn	RhoGAP19D	

ATPase-independent increased (164 genes)

AcCoAS	CG15673	CG3397	CG7530	drpr
Ald	CG15773	CG34336	CG7627	ebd2
Anxb11	CG15784	CG3788	CG7778	egh
Arc2	CG1673	CG3831	CG8051	Ets21C
ATPCL	CG17224	CG3967	CG8157	fax
baz	CG18249	CG42709	CG8547	for
Best1	CG18522	CG4587	CG8596	Gadd45
betaTub60D	CG2065	CG4928	CG8602	Gbs-76A
bnl	CG2991	CG5010	CG9098	Gclc
brm	CG30022	CG5224	CG9222	Gip
Cat	CG30089	CG5246	CG9629	Gpdh
CG10035	CG30345	CG5853	CG9663	GstD3
CG10433	CG30456	CG5958	CG9989	GstD4
CG10660	CG31075	CG6126	cnc	GstD5
CG10877	CG31694	CG6231	Cpr97Ea	GstD6
CG11727	CG31810	CG6330	Cyp6a17	GstD7
CG13248	CG32437	CG6424	Cyp6a20	GstD8
CG13250	CG32687	CG6776	Cyp6a22	GstD9
CG1358	CG32812	CG6785	Cyp6a23	GstE3
CG13795	CG32985	CG6954	Cyp6a9	GstE6
CG13907	CG33346	CG7056	Cys	GstE7
CG14085	CG33462	CG7299	dlt	GstE8
CG14340	CG3376	CG7460	DNaseII	GstE9

Hel89B	MRP	oys	scyl	TwdlE
Hex-A	Mrp4	Proct	Septin 4	Ugt86Da
Hk	MtnA	psd	smi35A	vfl
l(3)02640	Naam	Ptp4E	smp-30	vir-1
LamC	Neu3	pUf68	SP1173	wus
lcs	nimC4	Pvf1	Sp212	Xrp1
lectin-28C	Npc2a	Pvf3	sra	
LKR	NT5E-2	ref(2)P	Su(dx)	
Mct1	NTPase	Reg-2	Tim17a1	
mld	Oatp30B	RhoGAP93B	trol	
Mmp1	olf186-M	Sam-S	Tsp42Ef	

ATPase-independent decreased (106 genes)

Ack	CG1888	CG5151	HmgD	Rlip
Ama	CG30273	CG5726	Hml	sca
aru	CG31324	CG6175	hoe1	scramb1
bin3	CG32170	CG6472	InR	shn
br	CG32440	CG6490	Kaz1-ORFB	simj
brat	CG32486	CG7227	laza	Sk1
brk	CG32758	CG8788	lin-28	Smr
Cap-G	CG33307	chn	mex1	Snoo
CG10082	CG34417	Clic	mthl9	Spn
CG10543	CG3939	CtBP	N	srp
CG11138	CG4004	CTPsyn	Nckx30C	sty
CG11399	CG4019	daw	nemy	Su(z)2
CG12398	CG42394	Dp	Pat1	Sytbeta
CG12535	CG42566	Eaat1	Pepck	TfIIA-S-2
CG13741	CG42663	fd68A	PGRP-LE	tlk
CG13829	CG42846	Fit1	ph-p	Tre1
CG14291	CG42867	fray	pirk	wts
CG14478	CG42868	fru	pnr	yellow-f2
CG14812	CG43130	gce	PO45	
CG14879	CG43291	Hers	ppa	
CG15211	CG43759	hiw	Prestin	
CG17278	CG4927	HLHmbeta	Psc	

Table S2. List of 24 ATPase-independent genes differentially expressed in BRM-depleted cells

brm	CG12398	CG6954
Cat	CG6472	CG32440
Pepck	CG10433	CG33307
GstD3	lin-28	CG33462
Cyp6a9	CG9629	GstE3
chn	CG18249	CG42394
smi35A	CG3397	CG42846
CG15211	yellow-f2	CG43291

Table S3. List of enriched motifs in the promoters of BRM responsive genes
(with *E*-value < 9.9e-5)

MOTIFS ENRICHED IN ATPase-DEPENDENT GENES

Name	E-value	Region Center	Nr Matches	DNA-binding motif
Adf1	7,20E-09	88	154	MADF
Mad	8,50E-09	78.5	224	MAD homology
Hbn	2,00E-06	-141.5	180	Homeobox
Dbx	3,00E-06	-113.5	247	Homeobox
Engrailed	1,00E-05	-126.5	234	Homeobox
Ems	1,10E-05	-113	226	Homeobox
Ftz	1,50E-05	-138	204	Homeobox
Lim1	1,50E-05	-102.5	242	Homeobox, Zn-finger
Abd-A	1,80E-05	-118	254	Homeobox
H2.0	2,00E-05	-106	306	Homeobox
Apterous	2,30E-05	-110	290	Homeobox
Med	2,60E-05	97	182	SMAD, Dwarfin-type
AbdB	5,80E-05	-113.5	263	Homeobox
CG32532	8,60E-05	-130	179	Homeobox

MOTIFS ENRICHED IN ATPase-INDEPENDENT GENES

Name	E-value	Region Center	Nr Matches	DNA-binding motif
CG4328	2,30E-08	-114.5	265	Homeobox, Zn-finger
Exex	3,60E-08	-113	237	Homeobox
Mad	1,10E-07	77.5	213	MAD homology
Mip120	2,40E-07	-114	118	CRC
Dll	2,70E-07	-112	278	Homeobox
Lab	4,10E-07	-103	246	Homeobox
Hgtx	5,20E-07	-100.5	270	Homeobox
CG12361	9,20E-07	-112	250	Homeobox
Ubx	9,80E-07	-111	237	Homeobox
H2.0	1,00E-06	-115.5	253	Homeobox
Adf1	1,10E-06	95	168	MADF
NK7.1	1,30E-06	-111.5	256	Homeobox
Ems	3,10E-06	-124.5	200	Homeobox
Slou	4,80E-06	-112.5	220	Homeobox
Med	9,00E-06	108	200	SMAD, Dwarfin-type
Repo	9,50E-06	-103	242	Homeobox
Vsx2	1,30E-05	-111	247	Homeobox
AbdA	1,50E-05	-115	217	Homeobox
Dr	1,70E-05	-104.5	198	Homeobox
CG34031	1,90E-05	-113	265	Homeobox
E5	2,20E-05	-105.5	253	Homeobox
Dbx	2,40E-05	-108.5	239	Homeobox, helix-turn-helix
Lmx1a	2,40E-05	-106.5	177	Homeobox, Zn-finger
CG18599	3,20E-05	-104.5	192	Homeobox
Lms	3,30E-05	-113	165	Homeobox
Cad	3,50E-05	-108.5	234	Homeobox, helix-turn-helix
Ftz	3,80E-05	-107.5	240	Homeobox
Antp	4,00E-05	-112	242	Homeobox
BarH1	7,60E-05	-116.5	244	Homeobox
CG13424	7,90E-05	-113	179	Homeobox
Zen	8,30E-05	-142	161	Homeobox
Unpg	8,90E-05	-107	198	Homeobox

Table S4. Average fpmk values for selected transcription factors in control S2 cells and in cells that express recBRM proteins

ENSEMBL ID	Gene ID	Control	recBRM	recBRM-K804R
FBgn0000014	abd-A	0,02	0,00	0,01
FBgn0000015	Abd-B	0,00	0,03	0,01
FBgn0000054	Adf1	40,02	25,18	32,60
FBgn0260642	Antp	0,04	0,02	0,03
FBgn0000099	ap	432,34	197,70	315,02
FBgn0011758	B-H1	0,00	0,00	0,00
FBgn0000251	cad	0,16	0,03	0,09
FBgn0261723	Dbx	0,00	0,04	0,02
FBgn0034520	lms	0,00	0,00	0,00
FBgn0038592	CG18599	0,10	0,00	0,05
FBgn0052532	CG32532	0,21	0,07	0,14
FBgn0054031	CG34031	61,06	15,54	38,30
FBgn036274	CG4328	0,00	0,00	0,00
FBgn0000157	Dll	0,00	0,02	0,01
FBgn0000492	Dr	0,00	0,11	0,05
FBgn0008646	E5	0,00	0,00	0,00
FBgn0000576	ems	0,00	0,02	0,01
FBgn0000577	en	0,00	0,08	0,04
FBgn0041156	exex	0,04	0,13	0,09
FBgn0001077	ftz	0,00	0,00	0,00
FBgn0001170	H2.0	0,00	0,03	0,02
FBgn0008636	hbn	0,00	0,00	0,00
FBgn0040318	HGTX	0,00	0,03	0,02
FBgn0002522	lab	0,00	0,00	0,00
FBgn0026411	Lim1	0,00	0,03	0,02
FBgn0052105	Lmx1a	0,00	0,00	0,00
FBgn0011648	Mad	15,94	10,77	13,36
FBgn0011655	Med	5,81	5,46	5,63
FBgn0033846	mip120	14,83	17,84	16,34
FBgn0024321	NK7.1	13,20	6,63	9,91
FBgn0011701	repo	0,04	0,00	0,02
FBgn0002941	slou	0,02	0,02	0,02
FBgn0003944	Ubx	0,00	0,00	0,00
FBgn0015561	unpg	0,00	0,03	0,02
FBgn0263512	Vsx2	0,02	0,00	0,01
FBgn0004053	zen	0,00	0,00	0,00

Table S5. Sequences of PCR primers used for dsRNA synthesis

Brm BKNT7 F	taatacgactcactatagggagaAAGCCCAATCGCATTACAAC
Brm BKNT7 R	taatacgactcactatagggagaTGAACTGTATCAGCCGCTTG
Brm HFAT7 F	TTAATACGACTCACTATAGGGAGAgtttcgctgtacaataacaatc
Brm HFA T7 R	TTAATACGACTCACTATAGGGAGAatgtggagcaggacttaaag
GFP T7 F	taatacgactcactatagggagaATGGTGAGCAAGGGCGAGGAGCTG
GFP T7 R	taatacgactcactatagggagaGCGGTCACGAACTCCAGCAG

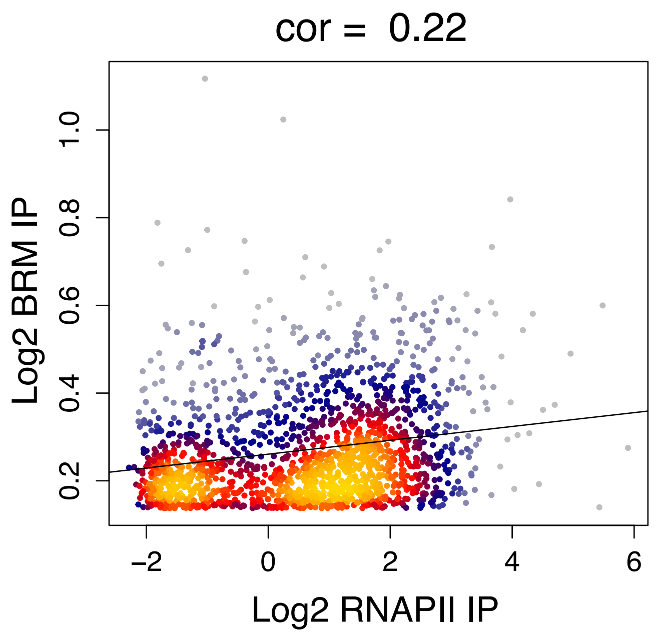

Figure S1. Comparison of input-corrected enrichment values for RNAPII (data from Lam et al. 2012) and BRM (this study) ChIP-seq datasets. The Pearson's correlation coefficient is indicated in the image. Colors indicate density of points, being yellow the highest density and grey the lowest.

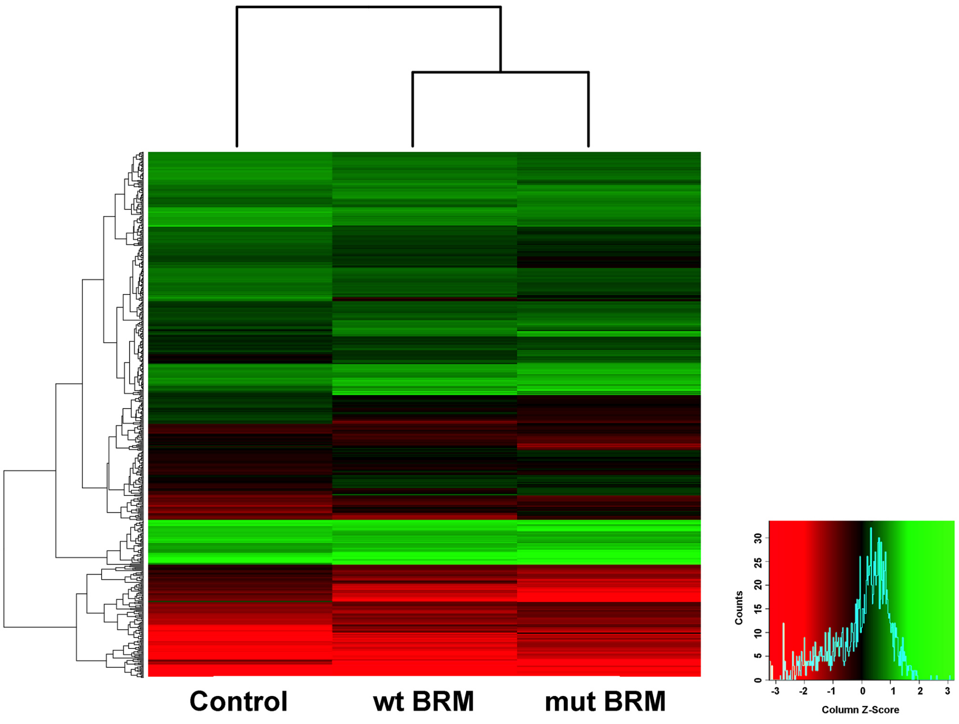

Figure S2. The heat map shows normalized gene expression of the BRM target genes in control cells and in cells that were depleted of endogenous BRM and expressed either wild-type BRM or mutant BRM.

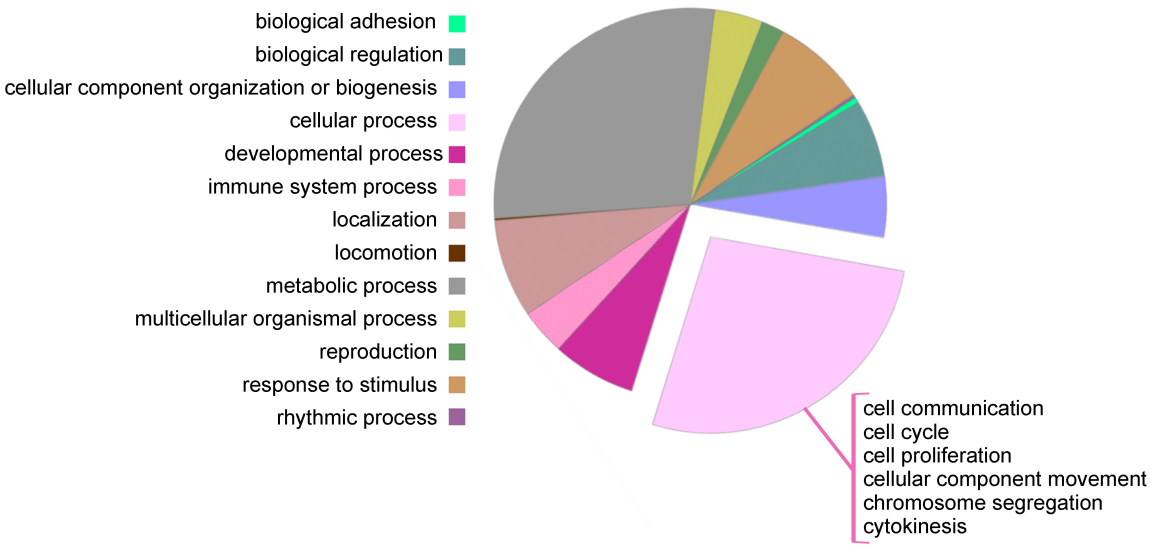

Figure S3. Gene ontology classification of the 541 BRM target genes identified in S2 cells by intersecting ChIP-seq and RNA-seq data. The analysis was carried out using Panther.

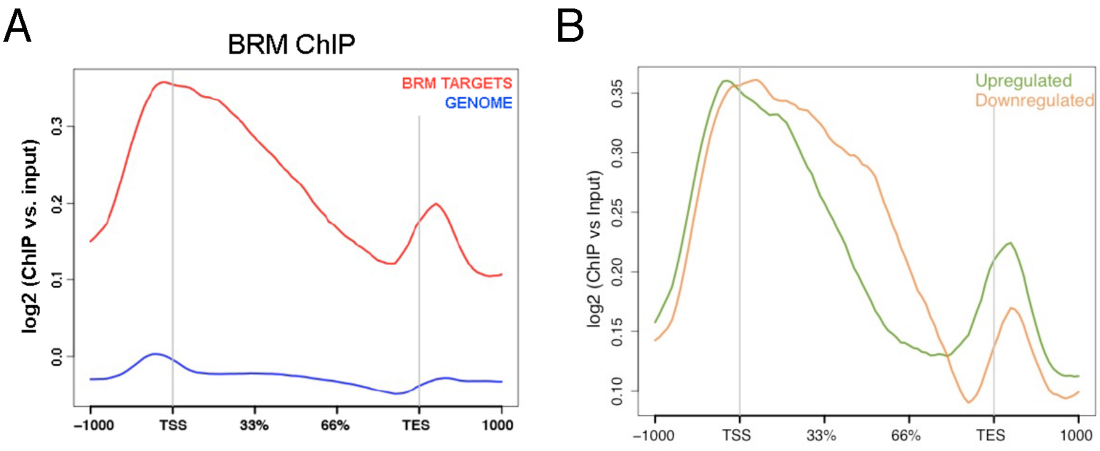

Figure S4. Metagene representations of BRM distribution. (A) The plot shows the distribution of BRM in the BRM target genes (red, n=541) and flanking sequences (X-axis) compared with the average distribution in all the S2 genes (blue, n=13294 genes). (B) The plot shows the distribution of BRM in the BRM target genes that were downregulated (brown, n=245) and upregulated (green, n=296) in cells that expressed recBRM. The difference in the gene body is statistically significant (K-S test, p=0.0175).

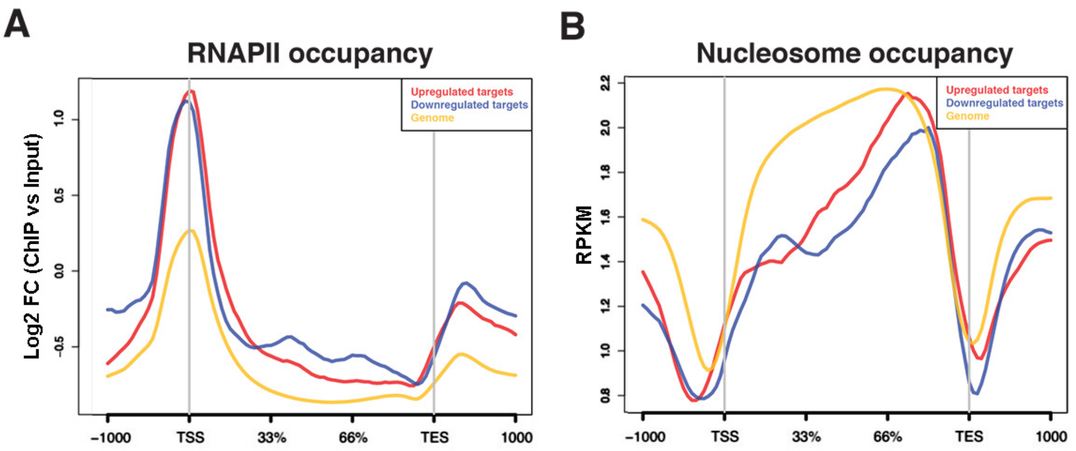

Figure S5. Metagene representations of RNAPII and nucleosome occupancies in BRM target genes upregulated or downregulated by BRM. The plots show the average distributions of RNAPII (A) and nucleosomes (B) in the BRM target genes that were downregulated (blue, n=245) or upregulated (red, n=296) in cells that expressed recBRM compared to control cells. The average distributions in all the S2 genes (yellow, n=13294 genes) are also shown for comparison.

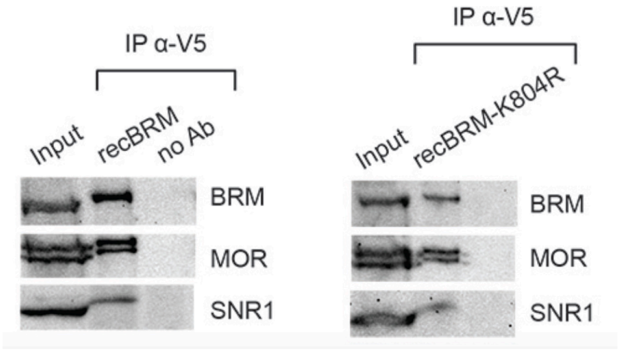

Figure S6. recBRM-V5 and recBRM-K804R-V5 are incorporated into SWI/SNF complexes. Immunoprecipitation experiments were carried out using an antibody against V5 in S2 cells that expressed either recBRM or recBRM-K804R. The input and the immunoprecipitated proteins were probed with antibodies against BRM, MOR and SNR1. The mobility differences between input and IP samples is likely due to different salt concentrations and to different total protein amounts.

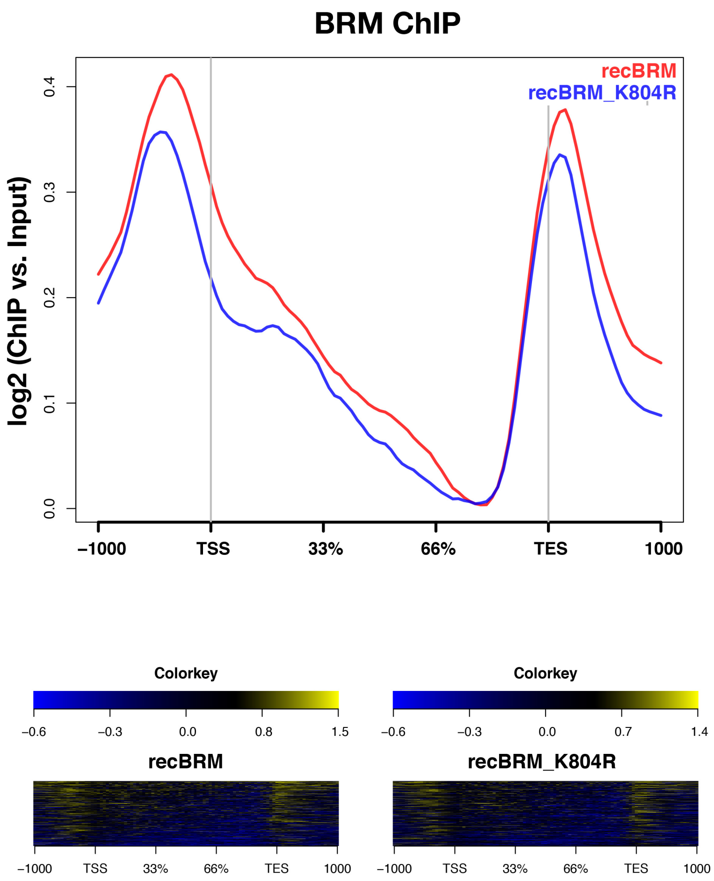

Figure S7. Genomic distributions of recBRM-V5 and recBRM-K804R-V5 analyzed by ChIP-seq using an anti-V5 antibody. The metagene shows average ChIP-seq signal for wild-type (red) and mutant (blue) BRM in the 541 BRM target genes. The heat maps in the bottom part of the image show V5 ChIP-seq signals over a region that includes the gene body, 1000 bp upstream of the TSS, and 1000 bp downstream of the TES.

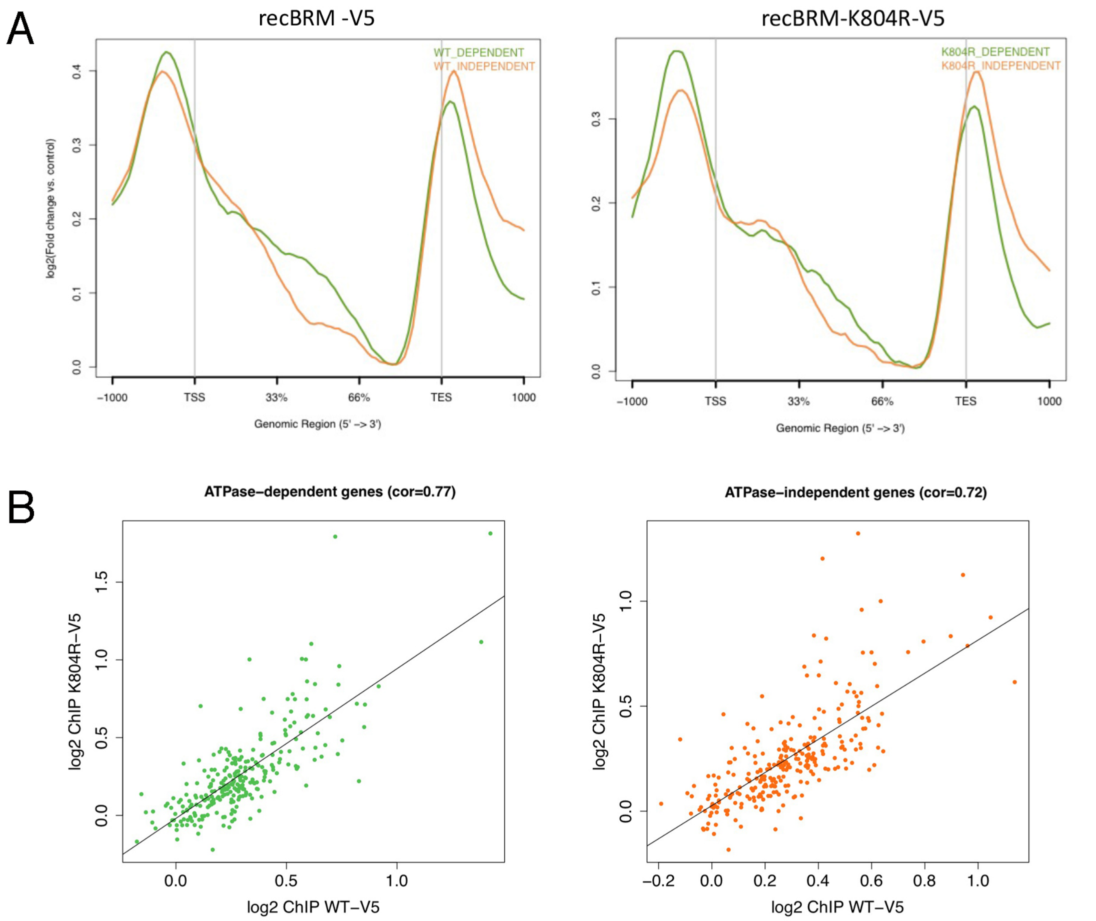

Figure S8. Distributions of recBRM-V5 and recBRM-K804R-V5 in ATPase-dependent (green) and ATPase-independent (brown) genes in S2 cells analyzed by ChIP-seq using an anti-V5 antibody. (A) The average distributions of active and mutant BRM were very similar to each other in both groups of genes. (B) The scatter plots show the levels of recBRM-V5 and recBRM-K804R-V5 for the individual BRM target genes. Pearson's correlations are indicated in the figure.

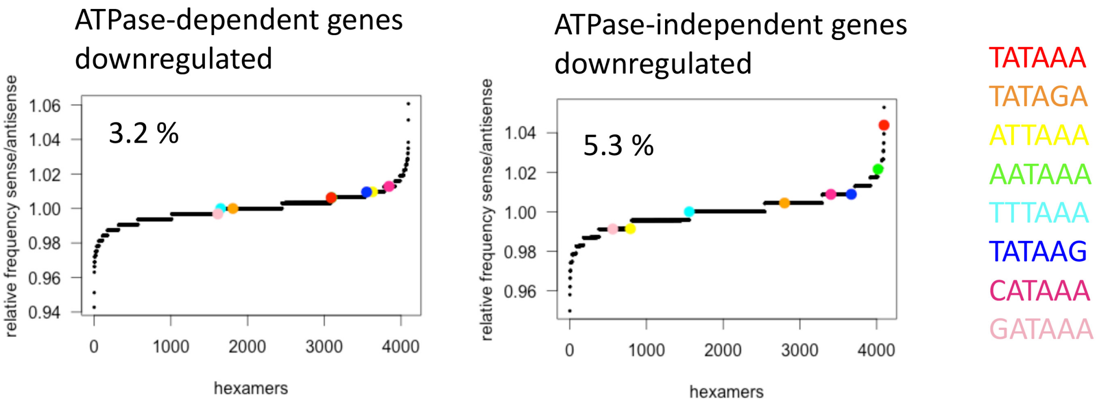

Figure S9. Analysis of TATA-box occurrence in the promoters of BRM target genes that are downregulated through ATPase-dependent (left, n=139 genes) and ATPase-independent (right, n=106 genes) mechanisms. The analysis was carried out as in Figure 5A.

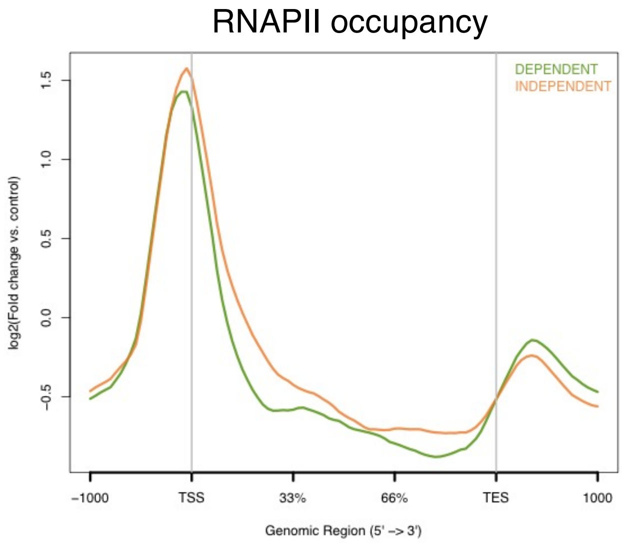

Figure S10. Metagene analysis of RNA polymerase II distribution in the ATPase-dependent (green) and ATPase-independent (brown) genes. The Y-axis gives the log2 of the ChIP signals normalized to input.

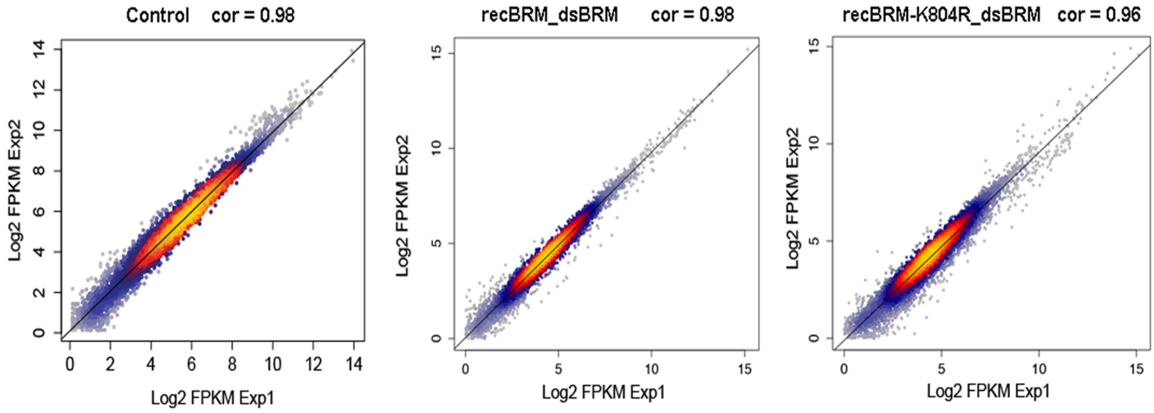

Figure S11. RNA-seq experiments: Pearson's correlation analysis of biological replicates.
